# Mucin 20 modulates proteasome capacity through c‐Met signalling to increase carfilzomib sensitivity in mantle cell lymphoma

**DOI:** 10.1111/jcmm.16953

**Published:** 2021-10-14

**Authors:** Xiaobin Wang, Fazal Shirazi, Wei Yan, Xiaoyu Liu, Hua Wang, Robert Z. Orlowski, Huihan Wang

**Affiliations:** ^1^ The Departments of Hematology Shengjing Hospital China Medical University Shenyang China; ^2^ The Departments of Lymphoma/Myeloma The University of Texas MD Anderson Cancer Center Houston Texas USA

**Keywords:** carfilzomib, c‐Met, mantle cell lymphoma, mucin 20, resistance

## Abstract

Mantle cell lymphoma (MCL) is a haematologic malignancy. The proteasome inhibitor (PI) bortezomib has been approved to treat MCL, but resistance has emerged through mechanisms that remain unclear. This study aimed to explore the mechanism of PI resistance in MCL and identify new targets for this patient subgroup. Carfilzomib‐resistant (CR) MCL cell lines and primary samples were used for both in vitro and in vivo experiments to identify gene expression and explore their related signalling pathways. We first identified mucin 20 (*MUC20*) suppression in carfilzomib‐resistant MCL models. MUC20 overexpression sensitized cells to carfilzomib in vitro and in vivo. *MUC20* expression was inversely related to activation of c‐Met and the downstream p44/42 MAPK pathway. c‐Met activation with hepatocyte growth factor (HGF) induced PI resistance, while c‐Met inhibition restored PI sensitivity. Carfilzomib resistance and depressed MUC20 expression were associated with enhanced proteasome activity and higher expression of proteassemblin (POMP), a chaperone for catalytically active proteasome assembly. c‐Met and POMP were associated through binding and induction of MAPK‐regulated ELK1 to the *POMP* promoter. Our data reveal that c‐Met signalling activation enhanced proteasome capacity as a mechanism of PI resistance, and *MUC20* expression may be a useful biomarker for PI therapy.

## 1 INTRODUCTION

Mantle cell lymphoma (MCL) is a haematologic malignancy that comprises 3%–6% of non‐Hodgkin's lymphomas. The clinical behaviour of MCL usually is aggressive, and its treatment strategy has gradually evolved to targeted therapy. Bortezomib is the first US Food and Drug Administration (FDA)‐approved proteasome inhibitor (PI) for treating newly diagnosed and relapsed/refractory MCL. In patients with relapsed or refractory MCL, bortezomib showed greater effectiveness compared to previous standard therapies and had a response rate of up to 50%.^1^, ^2^, ^3^ In previously untreated MCL,^4^ an improved median progression‐free survival (PFS, 24.7 months vs. 14.4 months) was observed in patients who received VR‐CAP (bortezomib, rituximab, cyclophosphamide, doxorubicin and prednisone) compared with R‐CHOP (rituximab, cyclophosphamide, doxorubicin, vincristine and prednisone). Bortezomib might also benefit patients with MCL as a maintenance therapy.^1^ However, primary and acquired resistance to bortezomib remains a major problem. Thus, current research is focused on identifying a new generation of PIs that can overcome bortezomib resistance.

Carfilzomib is a second‐generation PI that has received FDA approval for the treatment of myeloma. While bortezomib binds reversibly, carfilzomib is an epoxyketone that forms irreversible bonds, which can prolong the duration of proteasome inhibition. Carfilzomib is both tolerable and active against relapsed and/or refractory myeloma, even in some patients who have previously received bortezomib therapy.^5^, ^6^ The effect of carfilzomib for treating MCL is currently being explored. Zhang et al.^7^ used high‐throughput screening to test nearly 3800 approved drug candidates against MCL cell lines and identified four agents (alisertib, carfilzomib, pracinostat and YM155) across different therapeutic categories. These agents were found to have an antiproliferative effect in MCL cells, suggesting that carfilzomib is the agent that has the most potential to be effective. Wang et al.^8^ determined the therapeutic efficacy of carfilzomib in MCL both in vitro and in vivo. Carfilzomib‐induced apoptosis of MCL cells was mediated by the activation of JNK, Bcl‐2 and mitochondria‐related pathways. In addition, carfilzomib inhibited cell growth and survival through the NF‐κB and STAT3 signalling pathways. Zhang et al.^9^ also found that carfilzomib‐induced MCL cell apoptosis occurred in a caspase‐dependent manner through both intrinsic and extrinsic caspase pathways. In addition, carfilzomib inhibited constitutive activation of the NF‐κB signalling cascade in both MCL cell lines and primary MCL cells by completely blocking IκBα phosphorylation. Taken together, these data suggest that carfilzomib might be a potentially effective agent for treating MCL.

In this study, we aimed to explore the mechanism of PI resistance in MCL both in vivo and in vitro. Furthermore, we sought to identify a new targeted combinational therapy to improve the therapeutic effect of treatment for this subgroup of patients.

## 2 MATERIALS AND METHODS

### 2.1 Development of carfilzomib‐resistant (CR) cell lines

Drug‐naïve Granta‐519 and JeKo‐1 MCL cell lines were initially exposed to carfilzomib (Selleck Chemical) at 10% of the drug minimum inhibitory concentration (IC_10_). Over eight months, drug concentrations were serially increased from 1 to 10 nmol/L following the verification of proliferative ability. Once CR Granta‐519 and JeKo‐1 cell lines were established, all the experiments were performed after the cells were exposed to carfilzomib‐free medium for at least 7 days. All cultured cell lines had no microbial contamination.

### 2.2 Patient sample collection

Bone marrow samples were obtained from MCL patients admitted to the Shengjing Hospital of China Medical University. Informed consent was obtained from all patients, and the study was approved by the local ethics committee in accordance with the Declaration of Helsinki. Bone marrow mononuclear cells were isolated by Ficoll‐Hypaque density gradient centrifugation.

### 2.3 Cell viability assays

Cell viability assays were performed as described previously.^10^ Carfilzomib, bortezomib and tivantinib (ARQ‐197) were purchased from Selleck Chemical. The data provided were obtained from three independent experiments.

### 2.4 Real‐time reverse transcription‐polymerase chain reaction (RT‐PCR)

*MUC20* assay probes (Lot.1107622) were purchased from Applied Biosystems, and RT‐PCR was performed on an Applied Biosystems Step One Plus Real‐Time PCR system.

### 2.5 Flow cytometry

MCL CD20^+^ cells were analysed for MUC20 expression by multichannel flow cytometric analysis. Cells were incubated with monoclonal antibody (MAb) MUC20 (aa654‐684)‐PE (LS‐C223381) or mouse IgG1 as a control for 30 min. This process was followed by secondary labelling of the cells with PE‐conjugated goat anti‐mouse IgG for an additional 30 min. The cells were then incubated with APC‐conjugated anti‐CD20 MAbs and fixed in 2% paraformaldehyde. Stained cells were analysed by flow cytometry using FACScan and CellQuest Pro software (BD Biosciences).

### 2.6 Western blot

The antibodies used included anti‐MUC20 (PA5‐14973) from Thermo Fisher Scientific Pierce; anti‐phospho‐c‐Met (07–810) and ‐c‐Met (MAB3729) from EMD Millipore; anti‐phospho‐ERK1/2 (4370S), ‐ERK1/2 (9102S) and anti‐ELK1 (9182) from Cell Signaling Technology; and anti‐Proteassemblin (sc‐271414) and–H3 Histone (sc‐517385) from Santa Cruz Biotechnology. Western blot analysis was performed following standard procedures.^10^

### 2.7 Proteasome activity assays

Chymotrypsin‐like, trypsin‐like and caspase‐like activities were assayed using the Proteasome‐Glo™ Chymotrypsin‐Like, Trypsin‐Like and Caspase‐Like Cell‐Based Assay Kits (Promega).

### 2.8 Lentiviral vector technique and cell transfection

A *MUC20* lentiviral expression vector was prepared using pCDH‐CMV‐MCS‐EF1‐CopGFP (System Biosciences; Mountain View, CA, USA). The open reading frame of *MUC20* was PCR amplified from a cDNA clone (Origene; Rockville, MD, USA). Lentiviral particle‐infected cells were sorted by flow cytometry.

### 2.9 Luciferase reporter assays

The *POMP* promoter was amplified from genomic DNA, as follows: POMP‐p‐Kpn‐F, GGGGTACCCTAAGATGTCTCCATCCTGTGG and POMP‐p‐Bgl2‐R, GAAGATCTGTACCCACTCACCATCTTCCGCAGC. A KpnI and Bgl2 digest was then inserted into the pGl3‐basic vector. There are two ETS‐like gene 1 (ELK1) binding sites in the *POMP* promoter. We generated the front mutated (F‐mt) construct (CAGGACGGAC *GCACCAGTAA GGGA*TGTGGG GGCCAGCCCT C*GGAAACGGA AGTGA*GCGGC), the hind mutated (H‐mt) construct (CAGGACGGAC *GCACTTCCGG CGGA*TGTGGG GGCCAGCCCT *TCAGAATCTG AGTGA*GCGGC) and one in which both were mutated (2 mt). After constructs were transfected into HeLa cells using Lipofectamine 2000 (Life Technologies), luciferase activity was observed using the Dual‐Luciferase^®^ Reporter Assay System (Promega).

### 2.10 Electrophoretic mobility shift assays

The 5′ biotin‐labelled wild‐type (WT) probe sequence (wt‐F) was as follows: GACGGAC*GCACTTCCGGCGGA*TGTGGGGGCCAGCC CTC*GGAAACGGAAGTGA*GCGGC. The sequence spans the *POMP* transcription initiation site and includes the two *ELK1* sites. In the mutated *ELK1* site, the sequence (mut‐F) was as follows: GACGGACGCACCAGTAAGGGATGTGGGGGCCAGCCCTTCAGAATCTGAGTGAGCGGC. Protein‐DNA binding experiments were performed using the LightShift™ Chemiluminescent EMSA Kit (Thermo Fisher Scientific).

### 2.11 Chromatin immunoprecipitation

Chromatin immunoprecipitation was performed according to the manufacturer's protocol (EMD Millipore). The *POMP* promoter oligonucleotides used were as follows: F: CGCCGCTCACTTCCGTTT, R: TGGGTCCCTTGGGATTGC. The amplified product spanned the *POMP* transcription initiation site and included the *ELK1* binding sites.

### 2.12 Xenograft model

Granta‐519/CR cells and MUC20‐overexpressing Granta‐519/CR cells (5 × 10^6^/mouse) were separately subcutaneously xenografted into nude mice under a protocol approved by the Institutional Animal Care and Use Facility. Mice were treated with carfilzomib (5 mg/kg) or normal saline as a vehicle via intraperitoneal injection twice weekly. Tumour volume was calculated using the formula volume = (width)^2^ × length/2. The weight of the tumour was also determined. The mice were divided into the following four groups, which were injected with Granta‐519/CR or Granta‐519/CR/MUC20‐overexpressing (MUC20‐OE) cells and treated with vehicle or CFZ.

### 2.13 Statistical analysis

Statistical analyses were performed using unpaired *t* tests in GraphPad Prism, and *p*‐values less than 0.05 were deemed statistically significant.

## 3 RESULTS

### 3.1 Generation of CR MCL cells

**FIGURE 1:**
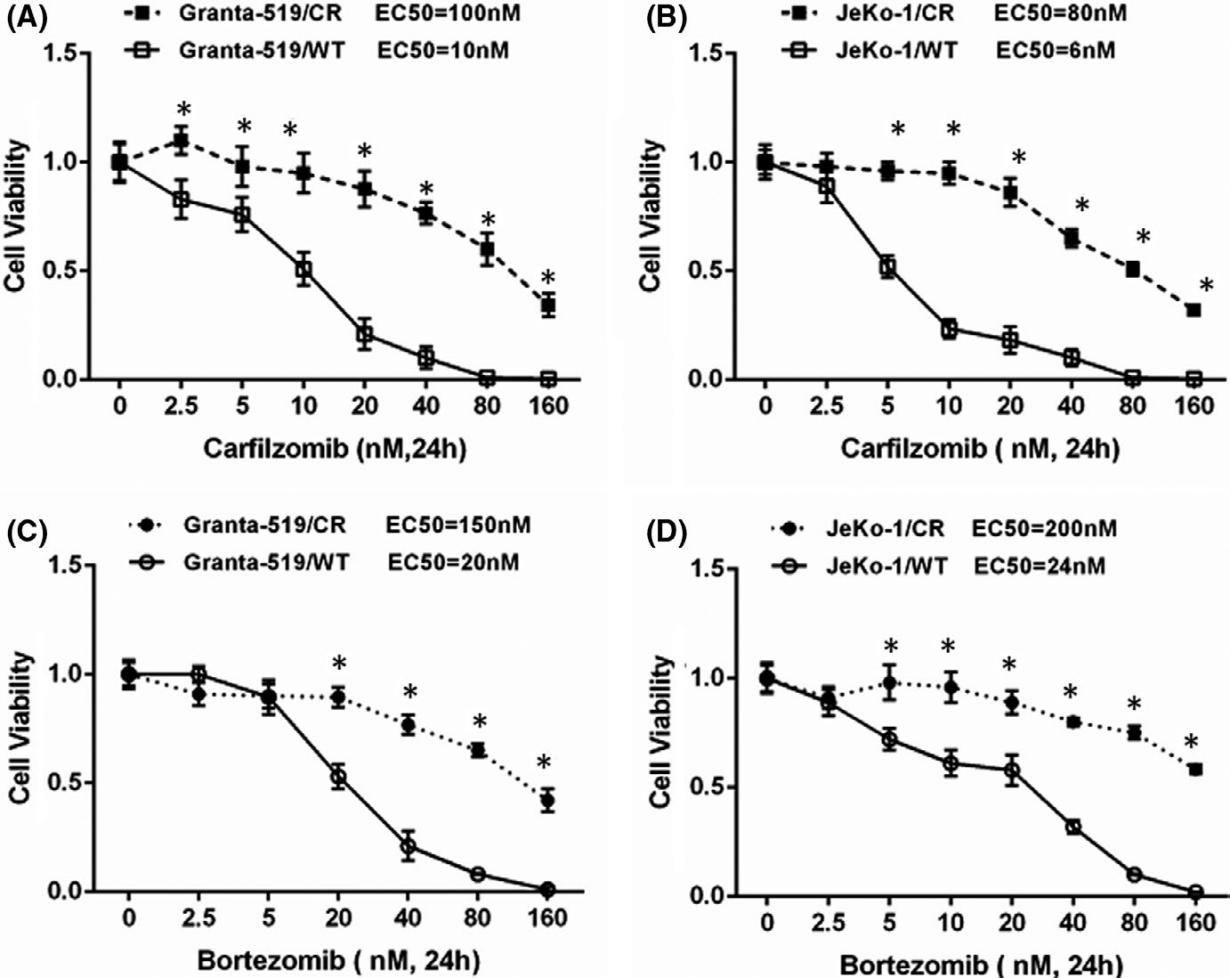
Resistance of mantle cell lymphoma (MCL) cell lines to both carfilzomib and bortezomib. (A) Viability of carfilzomib(CFZ)‐resistant (CR) MCL cell lines Granta‐519/CR and JeKo‐1/CR compared with their wild‐type (WT) counterparts. These cells were exposed to the indicated concentrations of carfilzomib or vehicle (0.1% DMSO) for 24 h, and viability was measured using the tetrazolium reagent WST‐1. *p < 0.05 when comparing CR cells to WT cells for all panels. (B) These same CR cells were then exposed to the indicated concentrations of bortezomib (BTZ) or vehicle and analysed as above

We generated Granta‐519 and JeKo‐1 cell lines to explore possible mechanisms of carfilzomib resistance. These phenotypes showed stable resistance to carfilzomib (Figure 1A). A similar pattern was also observed when these cells were exposed to bortezomib (Figure 1B). These findings suggested that some of the mechanisms of resistance in these cells were not carfilzomib‐specific and were applicable to other PI agents.

### 3.2 MUC20 expression decreased in MCL cells treated with PIs

**FIGURE 2:**
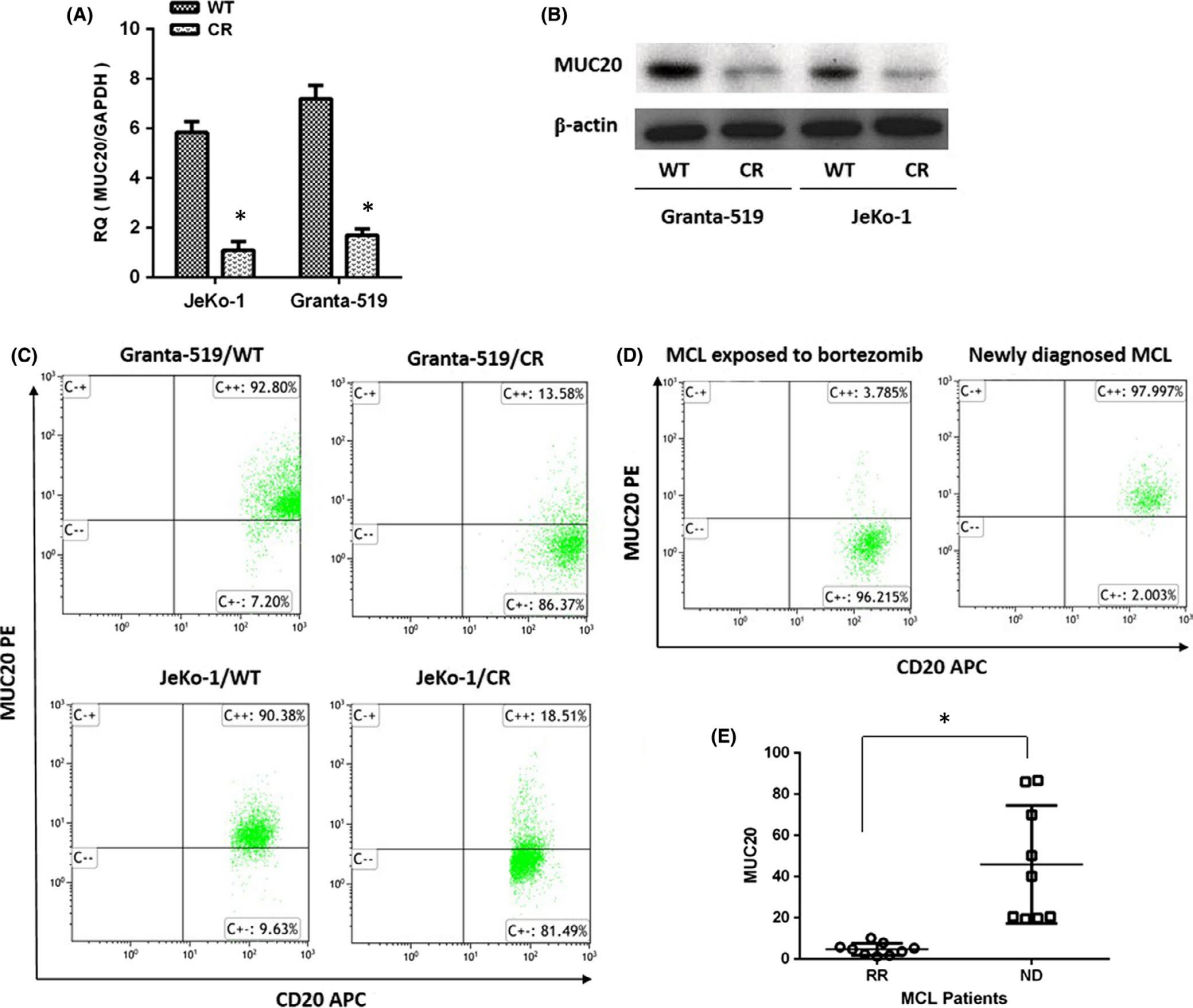
MUC20 expression decreased in mantle cell lymphoma (MCL) cells treated with proteasome inhibitors (PIs). (A) Real‐time polymerase chain reaction (RT‐PCR) analysis of *MUC20* mRNA content in carfilzomib‐resistant (CR) cells compared with their wild‐type (WT) counterparts. (B) Western blotting analysis of MUC20 protein levels in the WT and CR cell lines. (C) Flow cytometry analysis of membrane MUC20 expression in the WT and CR cell lines. (D, E) Flow cytometry analysis of MUC20 expression on CD20^+^ populations from the bone marrow (BM) of nine MCL patients (newly diagnosed and exposed to bortezomib). *p < 0.05. RR, relapse and resistance to bortezomib; ND, newly diagnosed

We found that *MUC20* expression was suppressed in CR myeloma cell lines. The GEP data were previously deposited in GEO bank with accession ID: GSE62237. We performed RT‐PCR to detect *MUC20* mRNA levels in CR MCL cells. The results showed a significant reduction in *MUC20* transcripts in CR (Figure 2A) MCL cells, which correlated with the reduced levels of MUC20 protein in CR (Figure 2B) cells observed via Western blot. Membrane MUC20 expression was also decreased in CR cell lines (Figure 2C) and in primary MCL samples exposed to bortezomib (Figure 2D, E). These data showed that there was varied MUC20 expression in WT and CR MCL cells, and MUC20 expression decreased when cells were treated with PIs.

### 3.3 MUC20 suppression correlates with PI resistance

**FIGURE 3:**
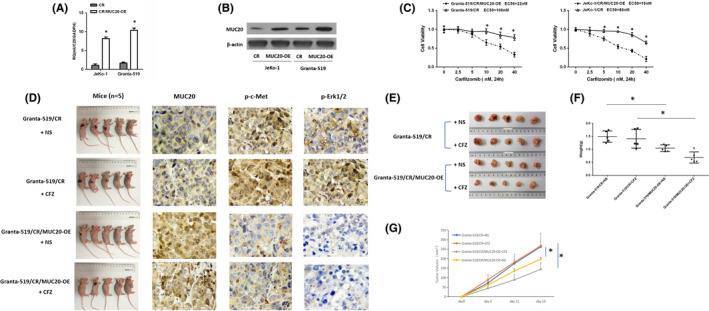
MUC20 expression correlates with proteasome inhibitor sensitivity. (A) Mantle cell lymphoma (MCL) cells were infected with cDNA, which led to MUC20 overexpression (MUC20‐OE). Real‐time polymerase chain reaction (PCR) detection of MUC20 mRNA and (B) Western blotting analysis of MUC20 protein levels to identify the infection effect. (C) Viability of CR cells compared with CR/MUC20‐overexpressing (MUC20‐OE) MCL cells in carfilzomib for 48 h. (D) Immunodeficient mice were randomly divided into four groups. Two groups were subcutaneously implanted with Granta‐519/CR cells and two were implanted with Granta‐519/CR/MUC20‐OE cells. One Granta‐519/CR group and one Granta‐519/CR/MUC20‐OE group were treated with 5 mg/kg CFZ by intraperitoneal injection twice a week. The other groups were treated with normal saline (NS) as a control. The expressions of MUC20, p‐c‐Met and p‐ERK1/2 in mice are shown. (E) Tumours in mice are shown. (F) The weight of tumours. (G) Tumour growth according to calliper measurement, which was calculated as the tumour volume using the equation (0.4 × L × W2). *p < 0.05

To determine whether MUC20 played a direct role in modulating PI sensitivity, we overexpressed MUC20 in CR cells (Figure 3A, B). When MUC20 was overexpressed in CR cells, sensitivity to carfilzomib (Figure 3C) was enhanced compared to that in the control. We also sought to validate these findings in a xenograft CR MCL model. Carfilzomib showed more activity in CR cells in the MUC20‐OE xenograft MCL model compared to in the CR xenograft model and MUC20‐OE xenograft MCL model treated with vehicle (Figure 3E). The MUC20‐OE mice had significantly lower tumour weight (Figure 3F) and volume (Figure 3G) compared with the other mice. MUC20 expression was not modified by CFZ therapy in MUC20‐overexpressing tumours. These data are consistent with the in vitro data, as presented in Figure S1. Together, these data support the notion that MUC20 expression levels are correlated with the sensitivity of MCL cells to PIs.

### 3.4 MUC20 suppression activates c‐Met signalling

**FIGURE 4:**
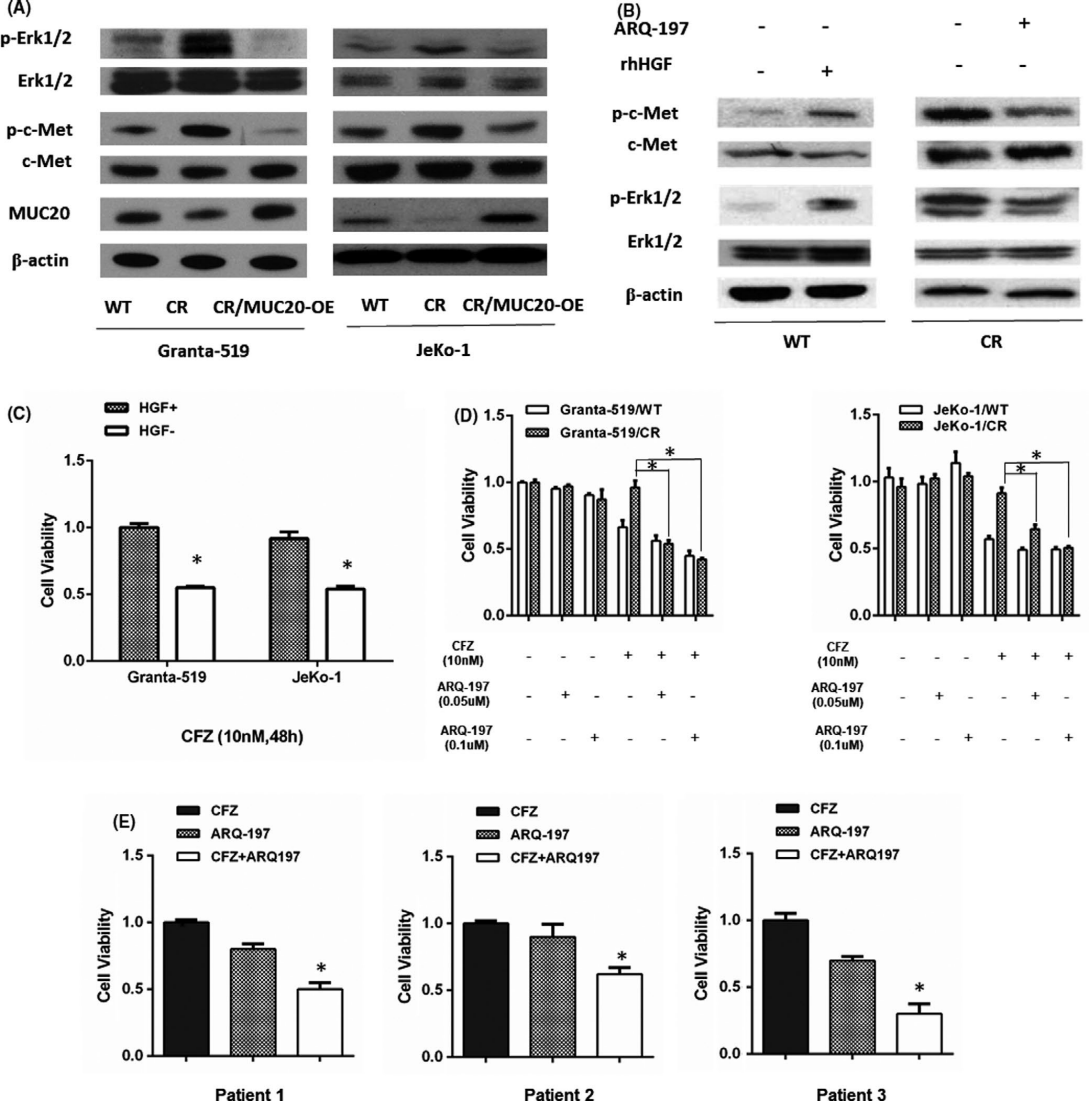
c‐Met signalling influences proteasome inhibitor sensitivity. (A) Extracts of Granta‐519 and JeKo‐1 carfilzomib‐resistant (CR) or wild‐type (WT) cells or CR cells overexpressing MUC20 (MUC20‐OE) were subjected to Western blotting to determine the activation status of MET and ERK. (B) Activation or inactivation status of c‐Met and ERK was determined when treated with recombinant human hepatocyte growth factor (rhHGF) or ARQ‐197 in Granta‐519 cells. (C) Viability of WT cells exposed to carfilzomib in the absence or presence of 100 ng/mL rhHGF for 24 h, according to WST‐1 assay. (D) Viability of WT and CR Granta‐519 and JeKo‐1 cells after exposure to CFZ with or without ARQ‐197 for 48 h. (E) Viability of primary MCL cell resistance to bortezomib after exposure to CFZ with or without ARQ‐197(0.1 µmol/L) for 48 h. *p < 0.05

The C‐terminus of MUC20 has been reported to bind a multifunctional docking site of the MET proto‐oncogene and suppress activation of some of its downstream signalling cascades.^11^ We therefore used Western blot to determine whether this mechanism could also be involved in the models used in this study. Granta‐519/CR and JeKo‐1/CR cells showed higher levels of MET and ERK1/2 activation than control cells (Figure 4A). Furthermore, MUC20 overexpression led to inactivity of MET and ERK1/2 (Figure 4A), and this phenomenon was also observed in the mouse xenograft model (Figure 3D). Since HGF is the activator of c‐Met signalling (Figure 4B), we explored whether HGF could antagonize the efficacy of carfilzomib. Indeed, HGF protected WT Granta‐519 and Jeco‐1 cells from the effects of carfilzomib (Figure 4C). These findings supported the hypothesis that c‐Met inhibition could restore the sensitivity of CR cells to carfilzomib. In our cell models, we found that the c‐Met inhibitor ARQ‐197 (tivantinib) (Figure 4B) did not reduce the viability of WT or CR Granta‐519 and Jeco‐1 cells when used alone at low concentrations (Figure 4D). However, enhanced activity was observed when cells were exposed to ARQ‐197 in combination with carfilzomib. Similar results were observed in MCL primary samples from patients who were exposed to and resisted bortezomib (Figure 4E). If the mechanism of carfilzomib resistance is through c‐Met signalling, this pathway could be an attractive clinical target.

### 3.5 c‐Met signalling impacts proteasome catalytic activity

**FIGURE 5:**
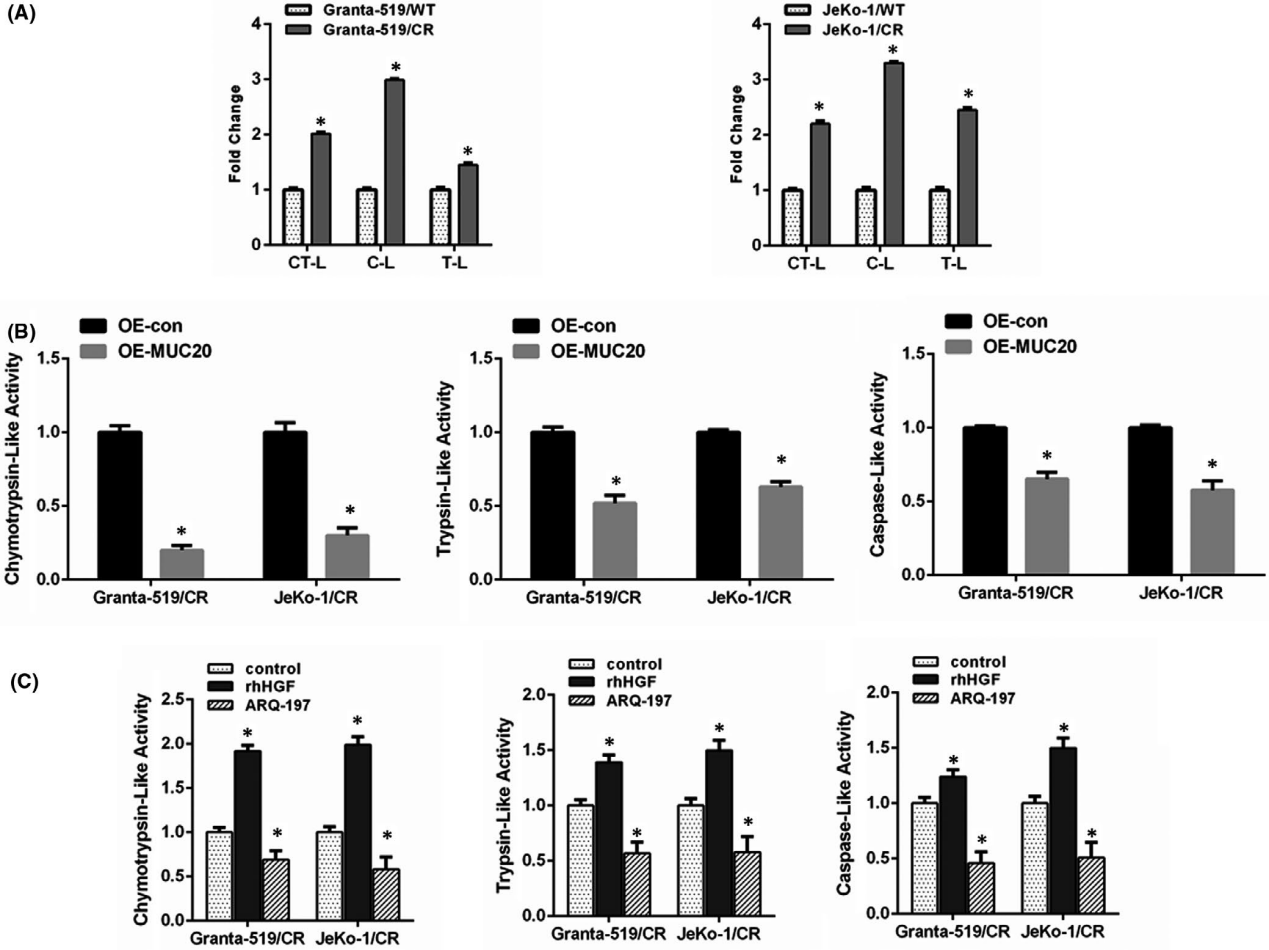
c‐Met signalling impacts proteasome catalytic activity. (A) The chymotrypsin‐like (ChT‐L), caspase‐like (C‐L), and trypsin‐like (T‐L) proteasome activity in wild‐type (WT) and carfilzomib‐resistant (CR) Granta‐519 and JeKo‐1 cells. (B) Proteasome activity in Granta‐519 and JeKo‐1 cells in which a vector or MUC20 had been overexpressed. (C) ChT‐L, T‐L and C‐L proteasome activity in CR Granta‐519 and JeKo‐1 cells treated with vehicle, 100 ng/mL recombinant human hepatocyte growth factor (rhHGF), or 1 μmol/L ARQ‐197 for 48 h. *p < 0.05 compared with the control group

Carfilzomib binds constitutive proteasome β5 subunit (PSMB5), which contains the chymotrypsin‐like activity (ChT‐L). Thus, we first examined ChT‐L activity in CR cells and found that ChT‐L activity was increased in CR Granta‐519 and JeKo‐1 cells compared to in their WT counterparts (Figure 5A). Moreover, the caspase‐like (C‐L) and trypsin‐like (T‐L) activities were also elevated in these two CR cell lines (Figure 5A). MUC20 overexpression in CR cells was itself sufficient to reduce proteasome activity (Figure 5B). Exposure of CR Granta‐519 and JeKo‐1 cells to HGF increased the ChT‐L, C‐L and T‐L activity, which further supports the relationship between c‐Met signalling and proteasome biology. Meanwhile, c‐Met signalling blockade using ARQ‐197 reduced the ChT‐L, C‐L and T‐L activity in CR cells (Figure 5C).

### 3.6 Proteassemblin (POMP) expression is linked to c‐Met activity

**FIGURE 6:**
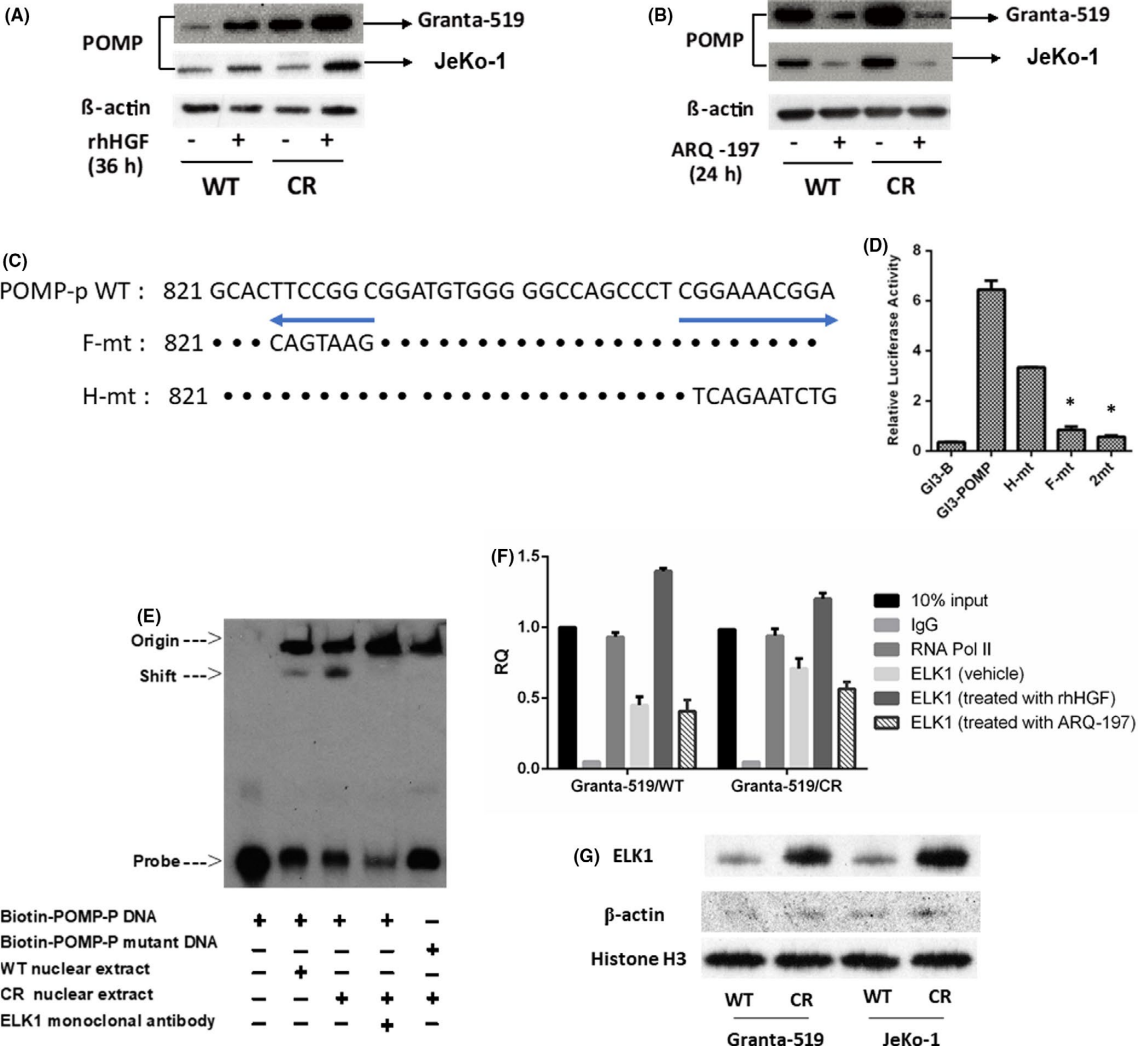
ELK1 regulates proteassemblin (POMP) expression and carfilzomib resistance. (A) The impact of 100 ng/mL exogenous human growth factor (HGF) for 36 h and (B) ARQ‐197 at 1 µmol/L for 24 h on POMP expression levels in wild‐type (WT) and carfilzomib‐resistant (CR) Granta‐519 and JeKo‐1 cells, as detected by Western blotting. (C) The sequences that were mutated to disrupt putative ELK1 binding sites in the POMP promoter. (D) Luciferase reporter assays showing the importance of the two ELK1 binding sites for POMP promoter activation in HeLa cells. These were transfected with constructs containing an empty luciferase promoter (GI3‐B) or a POMP promoter luciferase reporter with intact ELK1 sites (GI3‐POMP), one of the two ELK1 binding sites mutated (F‐mt and H‐mt), or (2 mt).” *p* < 0.05 vs. GI3‐POMP. (E) Electrophoretic mobility shift assay (EMSA) was performed using a 5′ biotin‐labelled oligonucleotide spanning the transcription initiation site of the POMP gene containing two ELK1 consensus binding sites (Biotin‐POMP‐P DNA). (F) Chromatin immunoprecipitation assays were performed in WT or CR cells using antibodies specific for ELK1. Nonspecific immunoglobulins (IgG) were used as a negative control, and antibodies to RNA polymerase II (RNA Pol II) were used as a positive control. The cells were pretreated with vehicle, 100 ng/mL rhHGF, or 1 μmol/L ARQ‐197 for 24 h. Quantitative real‐time polymerase chain reaction (PCR) analysis was used to observe the precipitation of POMP promoter sequences. The results were normalized to a 10% input of WT cells. (G) Nuclear ELK1 protein levels in CR and WT MCL cells, with Histone H3 protein as a loading control

The finding that all three major proteasome activities were increased in CR cells suggested that the assembly of subunits into functional proteasome particles was enhanced. POMP is the key chaperone responsible for assembly of the catalytically active beta subunit rings.^12^, ^13^ According to Western blot, POMP levels were increased in the CR Granta‐519 and JeKo‐1 cells compared to in WT cells (Figure 6A, B). Furthermore, HGF stimulation increased POMP expression in WT cell lines; this increase was also observed to some extent in the CR models (Figure 6A). This finding supports the connection between POMP and the c‐Met pathway. Meanwhile, pharmacologic c‐Met signalling blockade with ARQ‐197 reduced POMP levels in WT and CR cells (Figure 6B). Taken together, these data support a link between c‐Met pathway signalling and assembly chaperones that control proteasome capacity.

### 3.7 ELK1 regulates POMP gene expression

To elucidate the mechanism that linked c‐Met signalling and POMP, we examined transcription factor binding sites in the POMP promoter and found a consensus ELK1 binding site. This was of interest since ELK1 is a target for ERK1/2, which was found to be activated in CR cells. Western blotting demonstrated that nuclear extracts from CR cell lines were enriched for ELK1 protein (Figure 6G). To determine whether ELK1 indeed influenced *POMP* expression, we performed chromatin immunoprecipitation in CR cells using an anti‐ELK1 antibody, followed by PCR to detect sequences near the *POMP* promoter. While nonspecific IgG did not appreciably precipitate such sequences, they were comparatively enriched with anti‐ELK1 antibodies, and the enrichment was even greater in CR cells. In addition, HGF increased ELK1 binding to the *POMP* promoter, while ARQ‐197 decreased binding (Figure 6F). Next, we used a biotin‐labelled probe corresponding to the *ELK1* consensus site and nuclear extracts in a gel‐shift assay. We identified a protein‐DNA complex in WT cells, which was present in greater abundance when extracts from CR cells were used compared to WT extracts. Furthermore, an anti‐ELK1 antibody supershifted this band (Figure 6E). Since the *POMP* promoter has two consensus *ELK1* sites, we prepared *POMP* promoter reporter vectors that had one or both sites mutated (Figure 6C) and examined their activity in HeLa cells. The WT promoter was active under basal conditions compared to an empty vector Renilla luciferase reporter (GI3‐B). Notably, promoter activity was significantly decreased when either of the *ELK1* binding sites was mutated (H‐mt, F‐mt), and activity further decreased when both were mutated (2mt). F‐mt reduced luciferase activity almost as much as both mutations (F‐mt vs. 2mt), and H‐mt had much less of an effect (Figure 6D). These data suggest that the transcription factor ELK1 was downstream of the site of c‐Met pathway binding with the *POMP* promoter and increased POMP expression.

## 4 DISCUSSION

*MUC20* is a member of the mucin gene family, and prior research has suggested that its production is correlated with the progression of IgA nephropathy and other renal injuries.^14^ More recently, some studies have suggested a relationship between MUC20 and tumour pathobiology in other model systems. High MUC20 expression is considered a factor that negatively influences 5‐year cancer‐specific survival rates and recurrence‐free survival in early‐stage endometrial cancer patients.^15^ Another study showed that MUC20 was overexpressed and significantly correlated with recurrence and death in colorectal cancer.^16^ However, MUC20 has not yet been studied in the context of MCL, and its impact on its pathobiology and patient outcomes is unknown. In this study, we found that suppressed MUC20 expression was the most conserved and significant change in CR cells compared to in WT cells. Modulation of MUC20 expression levels using genetic approaches or pharmacologic agents targeting downstream effectors was sufficient to directly change PI sensitivity. Human *MUC20* is located close to *MUC4* on chromosome 3q29. *MUC4* has been implicated in the pathobiology of many human cancers^17^; furthermore, the expression and function of MUC4 in tumours vary widely and can indicate different outcomes.^18^, ^19^ Interestingly, a study found that MUC4 expression in pancreatic cancers is associated with enhanced sensitivity to bortezomib.^20^ Moreover, other studies have suggested that MUC1 may play a role in bortezomib resistance,^21^ and an anti‐MUC1 signal peptide vaccine has been explored in a Phase I/II study in multiple myeloma patients.^22^ Taken together, these data support the possibility that several members of the mucin family may be potential biomarkers for and mediators of the PI‐resistant phenotype. Thus, these members may be used as therapeutic targets for MCL treatment.

**FIGURE 7:**
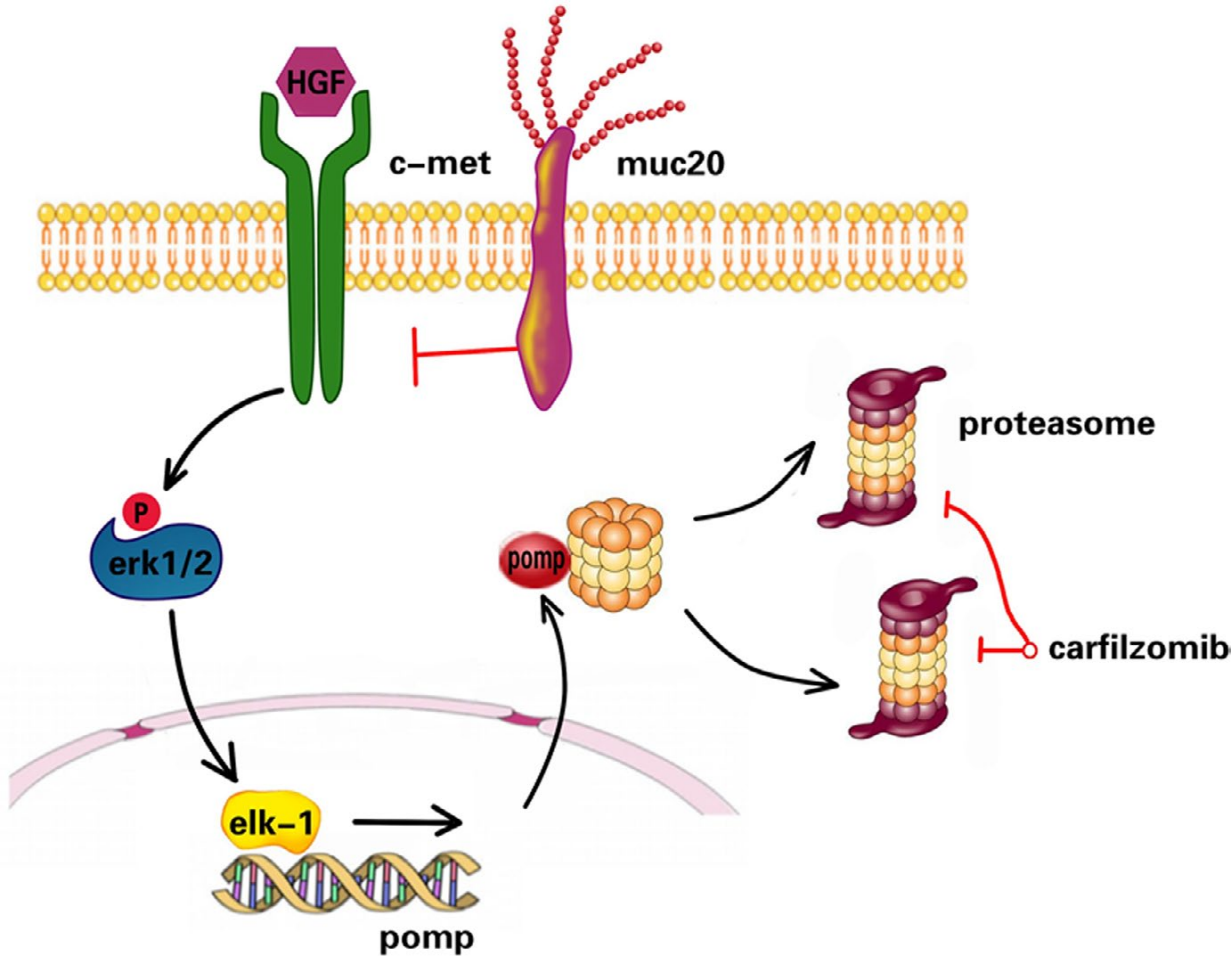
Schematic diagram of the MUC20/c‐MET/ERK/ELK1/POMP signal transduction pathway. Mantle cell lymphoma (MCL) cells expressing low MUC20 levels have high c‐Met/ERK/ELK1/POMP activity and high proteasome content and are therefore resistant to proteasome inhibitors

Studies of the effects of MUC20 expression have indicated that it may regulate the c‐Met signalling cascade. This seems to occur by decreasing HGF‐induced transient MAPK activation, blocking growth factor receptor‐bound protein (GRB)‐2 recruitment to MET, and suppressing the GRB2‐RAS pathway.^11^ HGF/c‐Met‐signalling is involved in a wide variety of human malignancies.^23^, ^24^, ^25^ However, compared to other solid tumours, there are limited data on the role of the HGF/c‐Met signalling pathway in lymphomas. HGF increased the adhesion of c‐Met‐positive B cell lymphoma cells to fibronectin and collagen that was mediated via β1‐integrin, which may suggest why HGF/c‐Met‐positive lymphomas have a poorer prognosis.^26^ HGF‐induced c‐Met activation in diffuse large B cell lymphoma (DLBCL) cells also leads to MEK‐dependent phosphorylation of the MAP kinases ERK1 and ERK2, which are associated with the regulation of cell proliferation.^27^ Natural killer/T cell lymphoma (NKTCL) cells were found to produce HGF and activate the HGF/c‐Met signalling pathway for tumour cell proliferation in an autocrine manner.^28^ In our study, we found that activation of c‐Met with HGF‐induced PI resistance, while c‐Met inhibition restored PI sensitivity. We also identified for the first time that increased expression of the β subunit ring assembly chaperone, POMP, is correlated with c‐Met activity. Our results show that *MUC20* expression may be used as a predictor of c‐Met pathway activity and PI sensitivity in MCL. Furthermore, our data suggest that loss of *MUC20* expression in the context of PI resistance could be a valuable tool to individualize MCL therapy. *MUC20* expression could be used to predicted whether patients would benefit from approaches that suppress the c‐Met pathway. In this study, we found that this decrease in *MUC20* expression reduces ERK1/2 and ELK1 signalling, inhibits *POMP* expression and reduces proteasome capacity, thereby restoring carfilzomib sensitivity (Figure 7). This was demonstrated in cell models using drugs that suppress c‐Met. More clinical trials using a combination of c‐Met inhibitors with PIs may be designed to determine whether the administration of this agent to patients clinically progressing on a carfilzomib‐based regimen can reverse this phenotype and restore PI drug sensitivity in MCL. Interestingly, MUC20 expression itself had no effect on Granta‐915 and JeKo‐1 cells in vitro (Figure S2). However, in the xenograft model, tumour growth was somewhat slower in MUC20‐OE mice than in CR models. These data suggested that another mechanism is involved in the reversal of CFZ resistance by MUC20, rather than affecting c‐Met signalling, and this phenomenon mostly occurs in vivo.

Our data reveal that enhanced proteasome capacity through c‐Met signalling activation is a mechanism of PI resistance. Furthermore, our results suggest that *MUC20* expression may be a useful biomarker in PI therapy.

## CONFLICT OF INTEREST

R.Z.O. has served on advisory boards for Onyx Pharmaceuticals and Takeda: The Millennium Oncology Company and received research support from these firms. The other authors have no conflict of interest to declare.

## AUTHOR CONTRIBUTIONS

**Xiaobin Wang:** Writing‐original draft (equal). **Fazal Shirazi:** Data curation (equal). **Wei Yang:** Data curation (equal). **Xiaoyu Liu:** Data curation (equal). **Hua Wang:** Data curation (equal). **Robert Z. Orlowski:** Conceptualization (equal); Investigation (equal); Supervision (equal); Writing‐review & editing (equal). **Huihan Wang:** Conceptualization (equal); Project administration (equal); Writing‐review & editing (equal).

## Supporting information

Figure S1

Figure S2

## Data Availability

Data sharing is not applicable to this article as no new data were created or analyzed in this study.
