# Correction to ‘Mucin 20 Modulates Proteasome Capacity Through c‐Met Signalling to Increase Carfilzomib Sensitivity in Mantle Cell Lymphoma’

**DOI:** 10.1111/jcmm.71186

**Published:** 2026-05-18

X. Wang, F. Shirazi, W. Yan, et al., “Mucin 20 Modulates Proteasome Capacity Through c‐Met Signalling to Increase Carfilzomib Sensitivity in Mantle Cell Lymphoma,” *Journal of Cellular and Molecular Medicine* 25, no. 21 (2021): 10164–10174, https://doi.org10.1111/jcmm.16953.

In Xiaobin Wang et al., the image for p‐c‐Met in ‘Granta‐519/CR+NS’ group of Figure 3D overlapped with p‐Erk1/2 in ‘Granta‐519/CR+CFZ’ group of Figure 3D due to the published error. The correct figure is shown below. The authors confirm all results and conclusions of this article remain unchanged.

**Figure:**
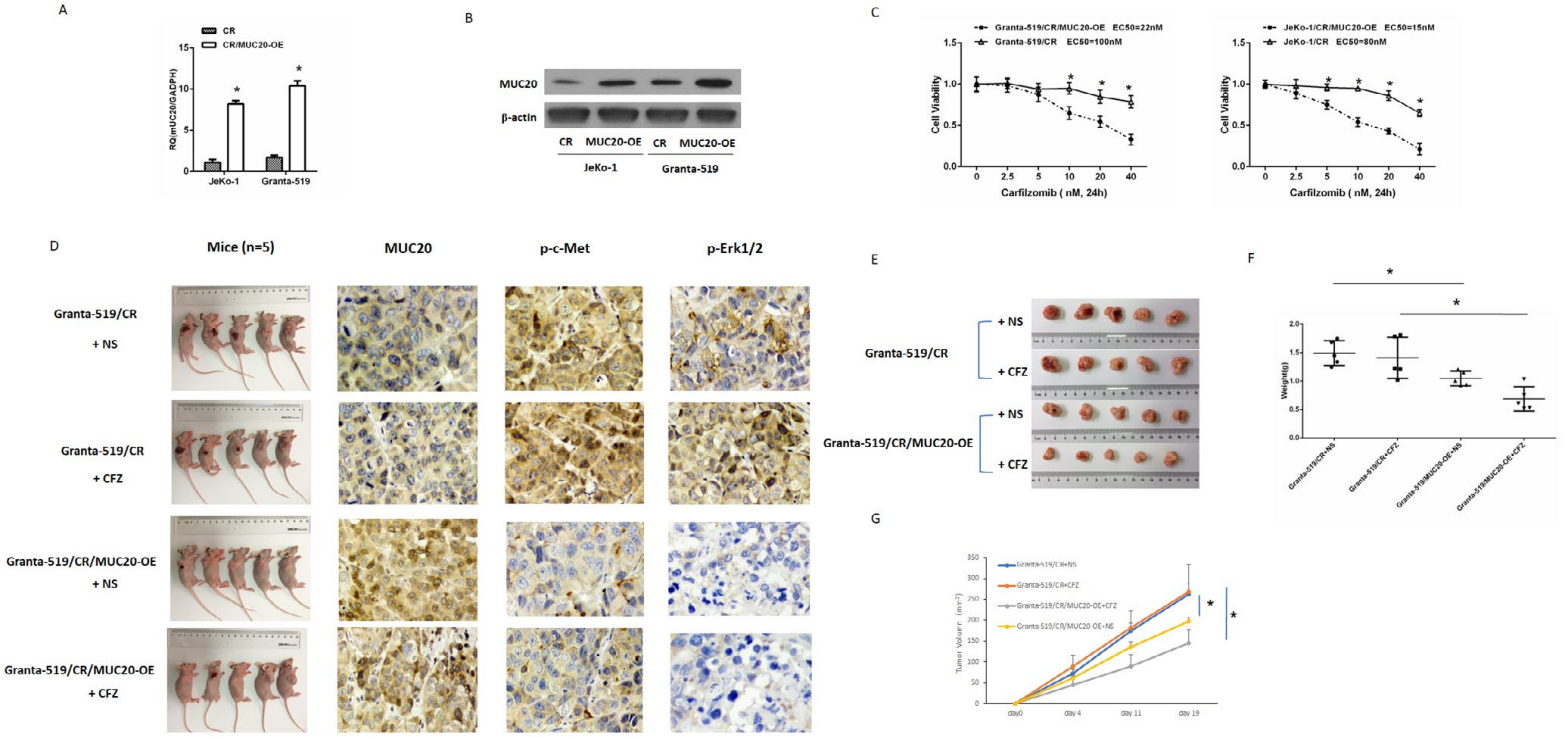
**FIGURE 3**. MUC20 expression correlates with proteasome inhibitor sensitivity. (A) Mantle cell lymphoma (MCL) cells were infected with cDNA, which led to MUC20 overexpression (MUC20‐OE). Real‐time polymerase chain reaction (PCR) detection of MUC20 mRNA and (B) Western blotting analysis of MUC20 protein levels to identify the infection effect. (C) Viability of CR cells compared with CR/MUC20‐overexpressing (MUC20‐OE) MCL cells in carfilzomib for 48 h. (D) Immunodeficient mice were randomly divided into four groups. Two groups were subcutaneously implanted with Granta‐519/CR cells and two were implanted with Granta‐519/CR/MUC20‐OE cells. One Granta‐519/CR group and one Granta‐519/CR/MUC20‐OE group were treated with 5 mg/kg CFZ by intraperitoneal injection twice a week. The other groups were treated with normal saline (NS) as a control. The expressions of MUC20, p‐c‐Met and p‐ERK1/2 in mice are shown. (E) Tumours in mice are shown. (F) The weight of tumours. (G) Tumour growth according to calliper measurement, which was calculated as the tumour volume using the equation (0.4 × L × W2). *p < 0.05.

