# Supplementary figures and images for: Mucin 20 modulates proteasome capacity through c‐Met signalling to increase carfilzomib sensitivity in mantle cell lymphoma
Source: J Cell Mol Med. 2021 Oct 14;25(21):10164–74. doi: 10.1111/jcmm.16953 (PMC8572801; doi:10.1111/jcmm.16953)

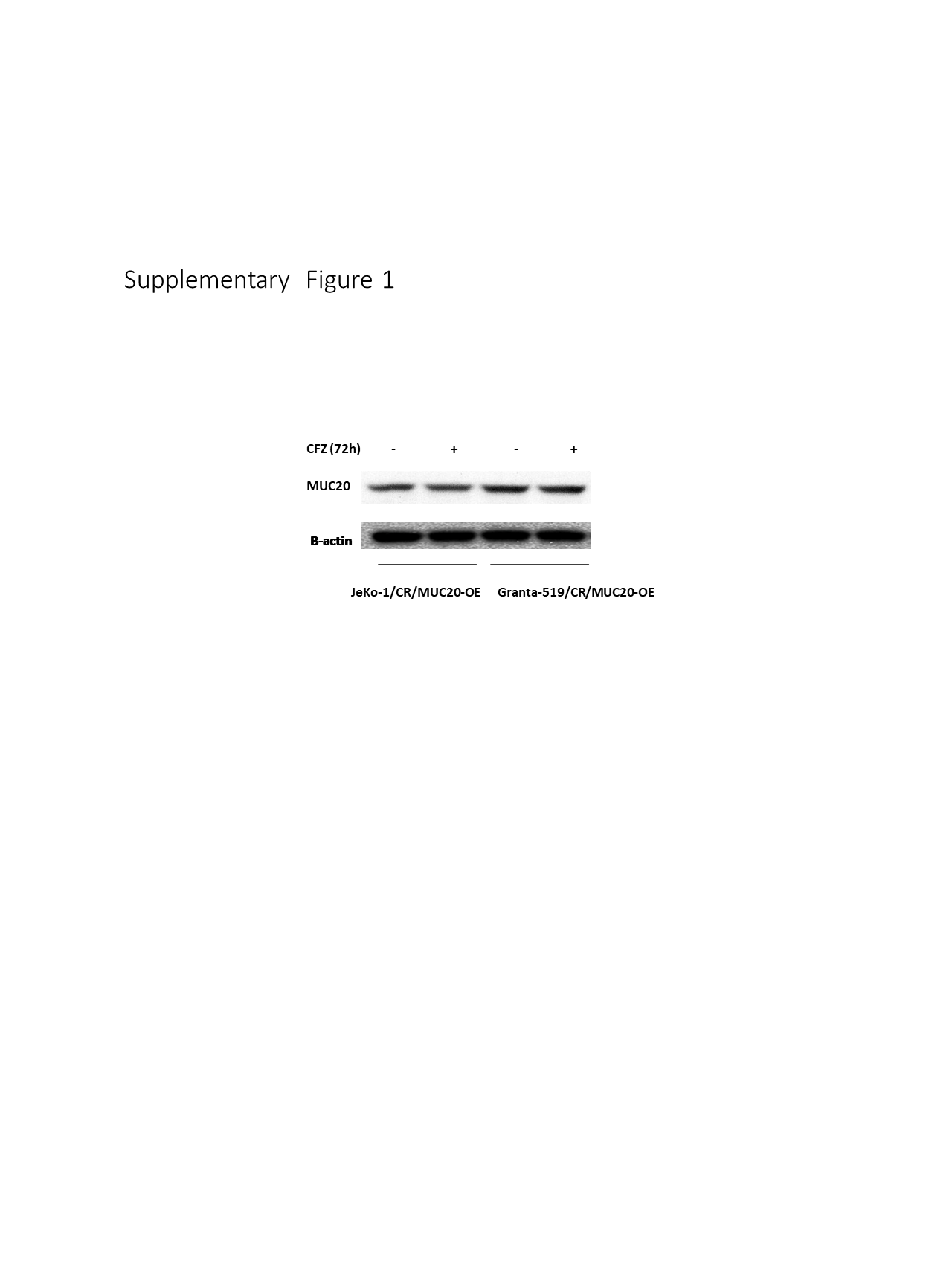

Supplement: Supplementary file 1 — Figure S1 [file JCMM-25-10164-s001.TIF]

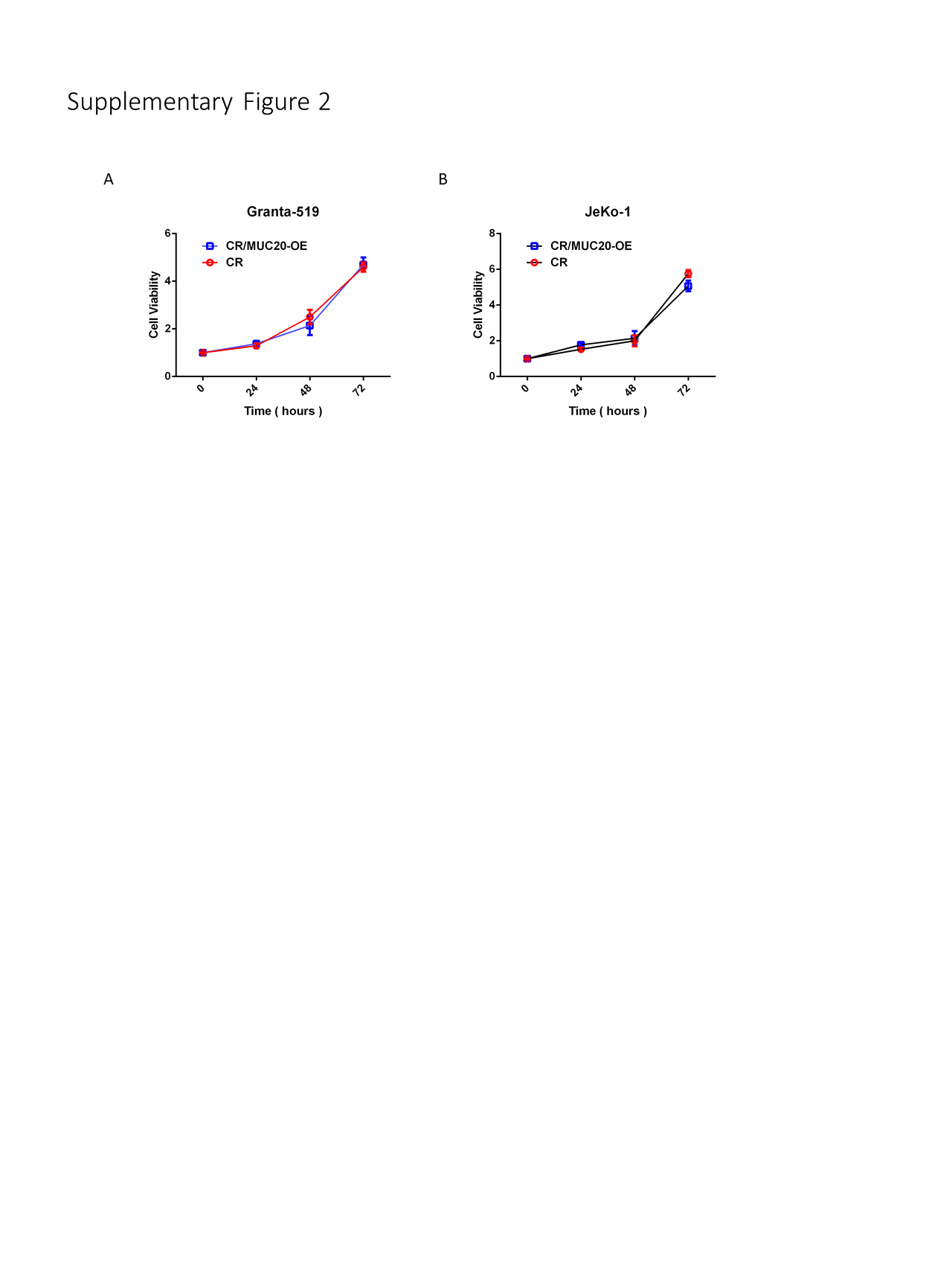

Supplement: Supplementary file 2 — Figure S2 [file JCMM-25-10164-s002.TIF]
